# Discrimination of *Lycium chinense* and *L. barbarum* Based on Metabolite Analysis and Hepatoprotective Activity

**DOI:** 10.3390/molecules25245835

**Published:** 2020-12-10

**Authors:** Min-Ji Ryu, Minjeong Kim, Moongi Ji, Chaeyoung Lee, Inho Yang, Seong-Bin Hong, Jungwook Chin, Eun Kyoung Seo, Man-Jeong Paik, Kyung-Min Lim, Sang-Jip Nam

**Affiliations:** 1 Department of Chemistry and Nanoscience, Ewha Womans University, Seoul 03760, Korea; ryumj624@naver.com; 2 College of Pharmacy, Ewha Womans University, Seoul 03760, Korea; nabe37@naver.com (M.K.); chaeyoung510@gmail.com (C.L.); yuny@ewha.ac.kr (E.K.S.); 3 College of Pharmacy, Sunchon National University, Suncheon 57922, Korea; wlansrl@naver.com; 4 Department of Convergence Study on the Ocean Science and Technology, Korea Maritime and Ocean University, Busan 4912, Korea; ihyang@kmou.ac.kr; 5 Biomix Co., Ltd. 142, Ilsan-ro, Ilsandong-gu, Goyang-si, Gyeonggi-do 10442, Korea; au4030@hanmail.net; 6 New Drug Development Center, Daegu Gyeongbuk Medical Innovation Foundation, Daegu 41061, Korea; jwchin@dgmif.re.kr

**Keywords:** *Lycium chinense*, *Lycium barbarum*, star pattern recognition analysis, gas chromatography-mass spectrometry

## Abstract

Lycii Fructus is a traditional medicine used to prevent liver and kidney diseases, which commonly derives from *Lycium chinense* and *Lycium barbarum*. Here, the extracts and ethyl acetate-soluble fractions of *L. chinense* fruits exhibited better hepatoprotective effects than those of *L. barbarum*, which was likely due to differences in their composition. Therefore, GC-MS and HPLC analyses were conducted to characterize the metabolite differences between *L. chinense* and *L. barbarum*. Based on amino acid (AA) and phenolic acid (PA) profiling, 24 AAs and 9 PAs were identified in the two species. Moreover, each species exhibited unique and readily distinguishable AA and PA star graphic patterns. HPLC analysis elucidated composition differences between the ethyl acetate-soluble layers of the two compounds. Further, NMR analysis identified their chemical structures as 4-(2-formyl-5-(hydroxymethyl)-1*H*-pyrrol-1-yl)butanoic acid and *p*-coumaric acid. The higher content of 4-(2-formyl-5-(hydroxymethyl)-1*H*-pyrrol-1-yl)butanoic acid was detected in *L. chinense*, whereas the content of *p*-coumaric acid was higher in *L. barbarum*. Therefore, the differences in the relative contents of these two secondary metabolites in the ethyl acetate-soluble layer of Lycii Fructus could be a good marker to discriminate between *L. chinense* and *L. barbarum*.

## 1. Introduction

Recent development of discrimination methods to distinguish herb products is mainly based on molecular evidence. For example, several molecular discrimination methods of the genus *Cynanchum* were developed employing InDel markers [1], the ARMS-PCR method [2], and ITS2 barcode analysis [3]. However, the discrimination methods based on molecular evidence are limited when a processed product is the target of the discrimination, which may lose the sequencing information during the extraction, heating, and drying procedures. From this perspective, chemical analysis may have its own merits and could be employed as a complementary method with a molecular analysis.

Lycii Fructus, the dried fruit of Barbary Wolfberry, is widely cultivated in China, Japan, and Korea due to its therapeutic value. Traditionally, Lycii Fructus was used as an herbal medicine to strengthen the liver and kidney [4], as well as an ingredient for functional teas in China, North America, and Southeast Asia [5]. Moreover, the uracil, rutin, and ascorbic acid contents of Lycii Fructus have been linked to anti-diabetic effects [6]. Cerebrosides and pyrrole compounds from this herb have been also reported to possess protective effects against hepatotoxicity caused by carbon tetrachlorides [7,8]. Betaine was also isolated from Lycii Fructus and was found to possess hepatoprotective, hypotensive, and anti-diabetic effects. However, betaine is a naturally occurring water-soluble *N*-timethylated amino acid both in *Lycium chinense* and *Lycium barbarum*. Therefore, it is not a suitable marker to discriminate between these two species [9,10]. Additionally, detailed studies are required to characterize the components of these *Lycium* species and their bioactivities. Therefore, GC-MS/HPLC-DAD was conducted to compare the metabolite profiles of *L. chinense* and *L. barbarum*. Amino acid (AA) and phenolic acid (PA) were analyzed by GC-MS, whereas secondary metabolites were analyzed by HPLC-DAD/LC-MS from the ethyl acetate (EA)-soluble extracts of both species. Our findings were ultimately used to discriminate between the two species.

## 2. Results

### 2.1. Hepatoprotective Activity of Extracts and Fractions of L. chinense and L. barbarum

**Figure 1:**
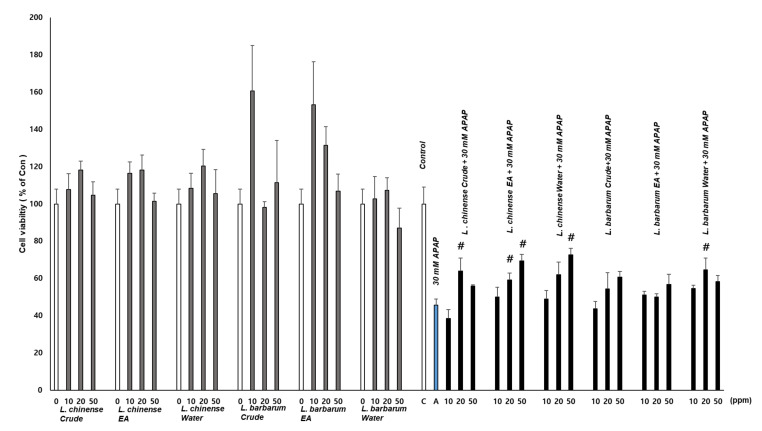
Effects of the *L. chinense* and *L. barbarum* crude extracts and fractions on HepG2 cell viability. The extracts were treated for 24 h with or without 30 mM acetaminophen and the resulting cell viability was measured via the WST-1 assay. *n* = 4 or more; # indicates a significant difference from the acetaminophen (APAP)-only group (*t*-test, *p* < 0.05).

The hepatoprotective effects of the crude extracts and fractions of *L. chinense* and *L. barbarum* were investigated in vitro. Hepatoprotective activities were evaluated against acetaminophen-induced cytotoxicity using HepG2 human hepatoma cells. The crude extracts and fractions of *L. chinense* displayed hepatoprotective effects. Specifically, the EA layer showed significant and dose-dependent activity. However, *L. barbarum* crude extracts had no effects, and the water layer showed weak activity at 20 ppm concentration only. As the crude extracts and fractions from both species showed no cytotoxicity on HepG2 cells, this difference suggested that the two species had different active compound compositions (Figure 1).

### 2.2. Amino Acid and Phenolic Acid Profiling Analyses

The presence of amino acids and phenolic acids of *L. chinense* and *L. barbarum* was analyzed using gas chromatography-mass spectrometry (GC-MS). A total of 24 amino acids (AAs) were identified as ethoxycarbonyl-*tert*-butyldimethylsilyl (EOC-TBDMS) derivatives (Table 1), and nine phenolic acids (PAs) were identified as *tert*-butyldimethylsilyl (TBDMS) derivatives (Table 2). The levels of AAs and PAs were reported as total (ng/mg) and normalized values in Table 1 and Table 2. Asparagine was the most abundant among the 24 aforementioned AAs in *L. chinense*, followed by proline and glutamine. In contrast, proline was the most abundant AA in *L. barbarum*, followed by glutamine and asparagine. Among the nine identified PAs, ferulic acid was the most abundant in *L. chinense*, followed by vanillic acid and 4-hydroxybenzoic acid, whereas, *p*-coumaric acid was the most abundant in *L. barbarum*, followed by ferulic acid and 4-hydroxybenzoic acid. The three most abundant AAs (asparagine, proline, and glutamine) in both species are known to possess liver-protective effects. The distribution of the three AAs varied greatly between the two species [11,12,13]. Additionally, the most abundant PAs (vanillic acid, *p*-coumaric acid, and ferulic acid) are known to have a hepatoprotective effect, and the three PAs also exhibited different distributions between the two species [14,15,16]. Therefore, among the AA and PA profiles of *L. chinense* and *L. barbarum*, these six metabolites were deemed suitable markers to distinguish between the two species.

### 2.3. Star Pattern Recognition Analysis

**Figure 2:**
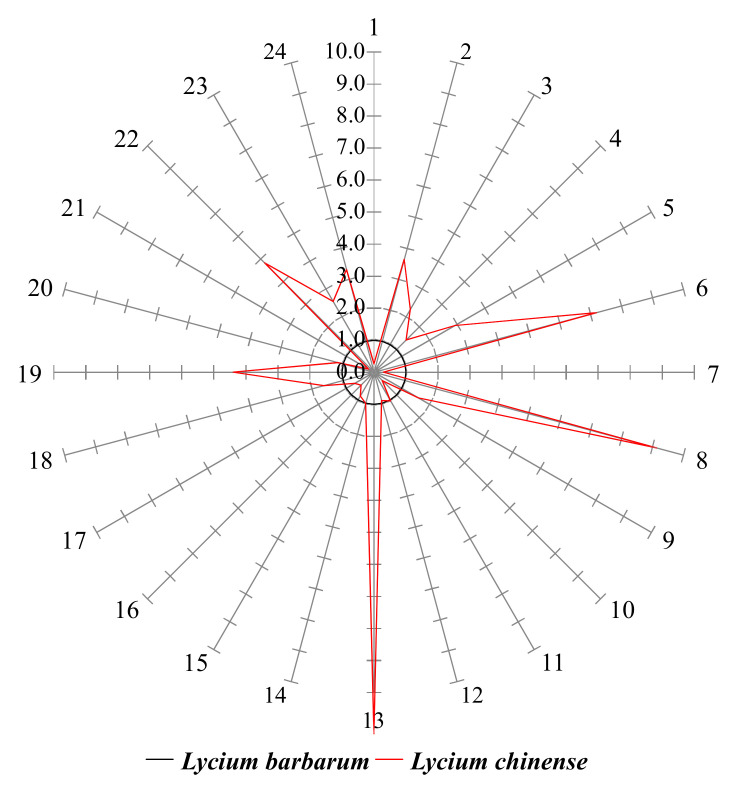
Amino acid star symbol plot of the normalized values in Table 1. The numbers on the rays correspond to the row no. in Table 1.

**Figure 3:**
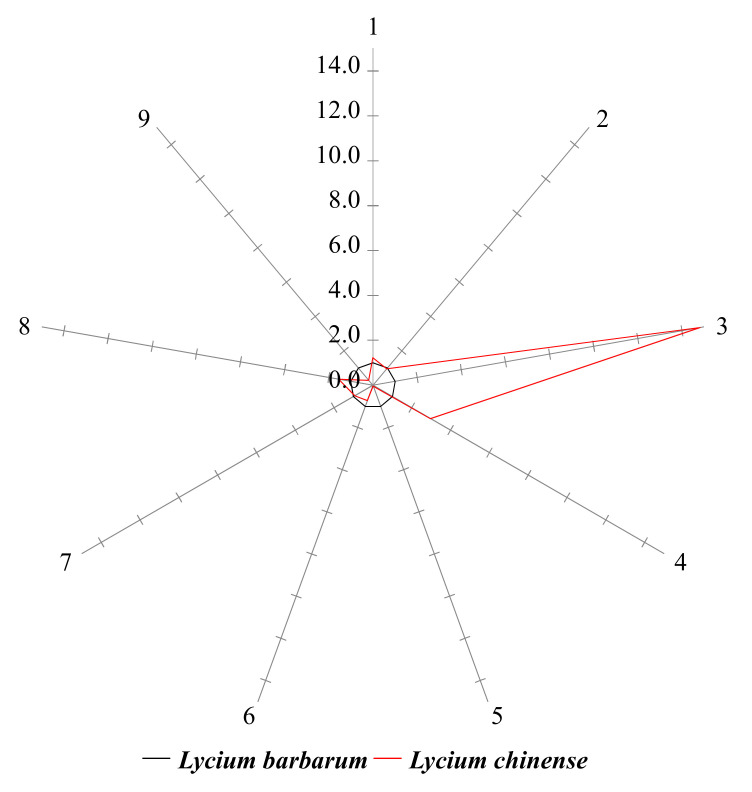
Phenolic acid star symbol plot of the normalized values in Table 2. The numbers on the rays correspond to the row no. in Table 2.

The levels of 24 AAs and 9 PAs in *L. chinense* were normalized to their corresponding mean levels in *L. barbarum* (Table 1 and Table 2). The overall patterns of AA and PA differences were informative to understand the differences between the two species. In the star symbol plot shown in Figure 2, the levels of eight AAs in *L. chinense* (alanine, proline, pyroglutamic acid, threonine, aspartic acid, *N*-methyl-DL-aspartic acid, homocysteine, and glutamine) were lower than those of *L. barbarum*. Moreover, the levels of 16 AAs were higher in *L. chinense* compared to those of *L. barbarum*. In the PA star symbol plots, the levels of five PAs in *L. chinense* were lower than those of *L. barbarum* (4-hydroxybenzoic acid, *p*-coumaric acid, protocatechuic acid, ferulic acid, and caffeic acid) (Figure 3). The differences between *L. chinense* and *L. barbarum* were clearly illustrated in the AA and PA star symbol plots (Figure 2 and Figure 3). Therefore, the star symbol plot patterns of AAs and PAs might be useful for the visual discrimination of *L. chinense* and *L. barbarum*.

### 2.4. Identification of Compounds with Different Contents in L. chinense and L. barbarum

**Figure 4:**
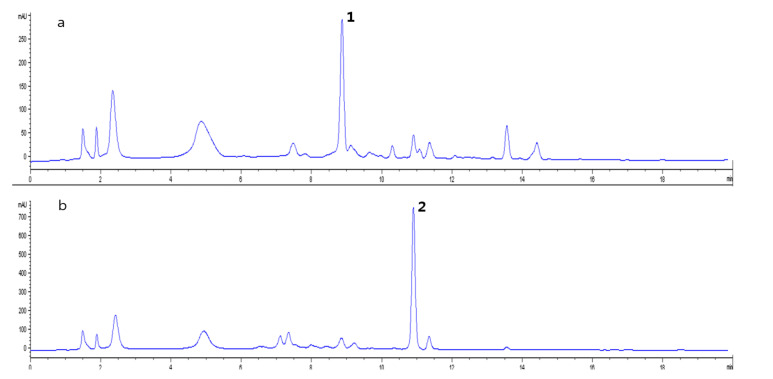
HPLC traces (detected at 310 nm) of (**a**) the ethyl acetate-soluble fraction of *L. chinense* and (**b**) *L. barbarum* extracts. These peaks were identified as 4-(2-formyl-5-(hydroxymethyl)-1*H*-pyrrol-1-yl)butanoic acid (**1**) and *p*-coumaric acid (**2**), respectively. The HPLC samples were prepared with 2 mg/mL of the extract by injecting 15 μL of each sample.

**Figure 5:**
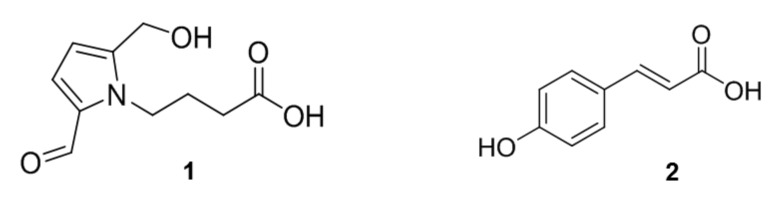
Chemical structures of 4-(2-formyl-5-(hydroxymethyl)-1*H*-pyrrol-1-yl)butanoic acid (**1**) and *p*-coumaric acid (**2**).

The ethyl acetate-soluble layers of *L. chinense* and *L. barbarum* were analyzed using HPLC. A peak with a retention time of 8.9 min in *L. chinense* was higher than that of *L. barbarum*, whereas a peak with a retention time of 10.9 min was higher in *L. barbarum* (Figure 4). HPLC-UV guided isolation of each extract yielded two compounds and their chemical structures were identified by comparing their spectroscopic data to those of previously reported compounds. The ^1^H-NMR spectroscopic data of a peak with a retention time of 8.9 min (**1**) displayed eleven proton signals, including an aldehyde proton (δ_H_ 9.43) and two heteroatomic aromatic protons (δ_H_ 6.98 (1H, d, *J* = 4.1 Hz, H-3) and 6.27 (1H, d, *J* = 4.1 Hz, H-4)). By comparing the ^1^H-NMR and MS data to those of previously characterized structures [8], compound **1** was identified as 4-[formyl-5-(hydroxymethyl)-1*H*-pyrrol-1-yl]butanoic acid (Figure 5). The ^1^H-NMR spectrum of a peak with a retention time of 10.9 min (**2**) showed para-substituted aromatic protons (δ_H_ 7.46 (2H, d, *J* = 8.6 Hz, H-2, H-6), 6.82 (2H, d, *J* = 8.6 Hz, H-3, H-5)) and two olefinic protons (δ_H_ 7.61 (1H, d, *J* = 15.8Hz, H-7), 6.30 (1H, d, *J* = 15.8 Hz, H-8)). The chemical structure of compound **2** was identified as *p*-coumaric acid based on previously reported ^1^H- and ^13^C-NMR data (Figure 5).

The contents of compounds **1** and **2** in 12 samples of two types of commercially available Lycii Fructus were analyzed. Compound **1** was detected in *L. chinense* only, whereas compound **2** was observed noticeably in *L. barbarum* (Figures S5 and S6 in Supplementary Materials). Thus, compounds **1** and **2** could be suitable markers to discriminate between *L. chinense* and *L. barbarum*.

### 2.5. Hepatoprotective Effect of 4-(2-formyl-5-(hydroxymethyl)-1H-pyrrol-1-yl)Butanoic Acid Isolated from L. chinense

Compound **1** (4-(2-formyl-5-(hydroxymethyl)-1*H*-pyrrol-1-yl)butanoic acid) is known to inhibit CCl_4_-induced glutamic pyruvic transaminase (GPT) release in rat primary hepatocytes. On the other hand, compound **2** (*p*-coumaric acid) is a well-known plant-derived natural product that reportedly inhibits melanin formation in murine melanoma cells stimulated by the α-melanocyte-stimulating hormone [17,18]. This compound also inhibits mushroom, murine, and human tyrosinases [19].

The hepatoprotective effect of the two herbs was evaluated against acetaminophen-induced cytotoxicity in HepG2 cells. *L. chinense* extracts and fractions exhibited strong hepatoprotective effects in a dose-dependent manner (Figure 1).

**Figure 6:**
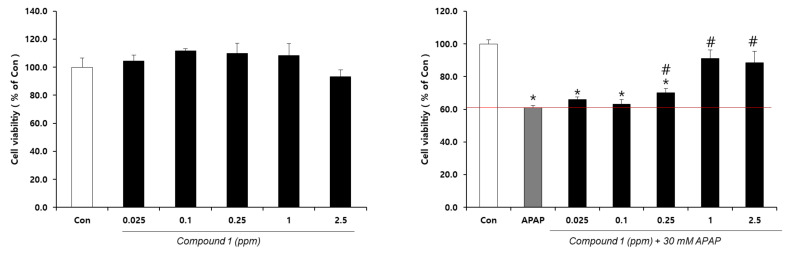
Effects of 4-(2-formyl-5-(hydroxymethyl)-1*H*-pyrrol-1-yl)butanoic acid (**1**) on the cell viability of HepG2 without (left) or with (right) 30 mM acetaminophen (APAP). *n* = 4 or more; # and * indicate significant differences from the APAP-only group and control, respectively (*t*-test, *p* < 0.05).

Further, the hepatoprotective effects of compound **1** were evaluated to confirm its putative properties. Notably, this compound exhibited dose-dependent and significant hepatoprotective effects up to 2.5 ppm (Figure 6).

## 3. Materials and Methods

### 3.1. Plant Material

One set of dried *L. chinense* and *L. barbarum* fruits were provided by Biomix Co. Ltd. (Goyang-si, Korea), whereas five sets were purchased from an herbal medicine commercial brand in Korea and were identified by one of the authors. A total six sets of dried *L. chinense* and *L. barbarum* fruits were used for analysis. After the analysis, the herb fruits were then each combined for the isolation of the compounds and prepared for the assay samples. Voucher specimens (ESN) documenting this purchase were deposited at the Natural Product and Medicine Research Laboratory, Department of Chemistry and Nanoscience, Ewha Womans University.

### 3.2. Chemical and Reagents

The AA standards, PA standards, ethyl chloroformate (ECF), and triethylamine (TEA) were purchased from Sigma-Aldrich (St. Louis, MO, USA). *N*-Methyl-*N*-*tert*-butyldimethylsilyl trifluoroacetamide (MTBSTFA) was obtained from Pierce (Rockford, IL, USA). Toluene, diethyl ether, ethyl acetate, and dichloromethane of analysis-grade pesticide residue were purchased from Kanto Chemical (Tokyo, Japan).

### 3.3. Extraction, Fractionation, and Isolation of Compounds

The dried and ground *L. chinense* (0.8 kg) and *L. barbarum* (0.8 kg) fruits were separately extracted with distilled water (3 L) via 30 min of sonication (40 kHz frequency, 523 W, DAIHAN, Seoul, Korea) and then the water (0.1 L) was removed in vacuo to obtain the crude extracts. The rest (2.9 L) was then partitioned with ethyl acetate (EtOAc) (5 L × 4 times). The water-soluble layer was dried in vacuo with large-capacity evaporator WEV-1020 (DAIHAN, Seoul, Korea) to have the water extracts of *L. chinense* (45 g) and *L. barbarum* (36 g), respectively. The EtOAc-soluble layer was dried in vacuo with the large-capacity evaporator. After filtration and concentration, the EtOAc extract (2.1 g) of *L. chinense* was fractionated via silica normal phase column chromatography eluting with a step gradient from 0 to 100% methanol (MeOH) in dichloromethane (DCM) distributed into eight fractions. The sixth fraction (DCM:MeOH = 80:20, 158 mg) was subjected to reversed-phase HPLC with 40% aqueous acetonitrile (Watchers 120 ODS-BP, ISU Industry Corp., Seoul, Korea, 250 × 10 mm, 5 μm, 2.0 mL/min, UV = 310 nm) to render 4-(2-formyl-5-(hydroxymethyl)-1*H*-pyrrol-1-yl)butanoic acid (**1**, 2.0 mg) with a retention time of 8.9 min. The EtOAc extract (1.8 g) of *L. barbarum* was fractionated by silica normal phase column chromatography eluting with the same ratio of DCM and MeOH. The fourth fraction (DCM:MeOH = 95:5, 112 mg) was subjected to reversed-phase HPLC with 23% aqueous acetonitrile (Watchers 120 ODS-BP, 250 × 10 mm, 5 μm, 2.0 mL/min, UV = 280 nm) to render *p*-coumaric acid (**2**, 13.4 mg) with a retention time of 10.9 min. The isolation of the compounds was performed by a Waters 616 quaternary HPLC pump and a Waters 996 photodiode array detector (Waters, Milford, MA, USA).

*4-[Formyl-5-(hydroxymethyl)-1H-pyrrol-1-yl]butanoic acid* (**1**): ^1^H-NMR (400 MHz, CD_3_OD) δ_H_ 9.43 (1H, s, CHO), 6.98 (1H, d, *J* = 4.1 Hz, H-3), 6.27 (1H, d, *J* = 4.1 Hz, H-4), 4.65 (2H, s, H-6), 4.39 (2H, t, *J* = 7.6 Hz, H-1′), 2.31 (2H, t, *J* = 7.5 Hz, H-3′), 2.01 (2H, q, *J* = 7.4 Hz, H-2′); ^13^C-NMR (100 MHz, CD_3_OD) δ_C_ 180.9 (CH, C-1), 176.7 (qC, C-4′), 144.6 (qC, C-5), 133.5 (qC, C-2), 126.4 (CH, C-3), 111.5 (CH, C-4), 56.4 (CH_2_, C-6), 45.8 (CH_2_, C-1′), 31.7 (CH_2_, C-3′), 27.6 (CH_2_, C-2′); LRMS: *m*/*z* 212.3 [M + H]^+^.

*p-Coumaric acid* (**2**): ^1^H-NMR (300 MHz, CD_3_OD) δ_H_ 7.61(1H, d, *J* = 15.8Hz, H-7), 7.46 (2H, d, *J* = 8.6 Hz, H-2, H-6), 6.82 (2H, d, *J* = 8.6 Hz, H-3, H-5), 6.30 (1H, d, *J* = 15.8 Hz, H-8); ^13^C-NMR (125 MHz, CD_3_OD) δ_C_ 170.0 (qC, C-9), 160.0 (qC, C-4), 145.3 (CH, C-7), 130.8 (CH, C-2, C-6), 126.6 (qC, C-1), 116.7 (CH, C-3, C-5), 115.9 (CH, C-8); LRMS: *m*/*z* 165.0 [M + H]^+^.

### 3.4. Hepatoprotection Assay

HepG2 cells (human hepatocellular carcinoma) were obtained from ATCC (Manassas, VA, USA). The cells were cultured in 75T culture plates and were maintained in standard culture conditions using Dulbecco’s Modified Eagle’s Medium (DMEM) containing 10% fetal bovine serum (FBS), 1% penicillin-streptomycin, and incubated in a 5% CO_2_ humidified atmosphere at 37 °C [20,21,22,23,24,25,26]. The culture medium was changed twice a week. At 70 to 80% cell confluence, adherent cells were released with a solution of trypsin (Hyclone, South Logan, UT, USA). HepG2 cells were seeded at 1 × 10^6^ cells/mL for the experiments. WST-1 (4-[3-(indophenyl)-2-(4-nitrophenyl)-2*H*-5-tetrazolio]-1,3-benzene disulfonate) (Roche, Indianapolis, IN, USA) solution was used to investigate cell viability. For the WST-1 assay, 1 × 10^4^ cells were seeded into 96-well plates. HepG2 cells treated with the samples (with or without acetaminophen) were incubated with 200 μL of WST-1 solution for 2.5 h at 37 °C at 5% CO_2_ in the dark. A total of 100 μL of the supernatant was transferred into each well of a 96-well plate, and absorbance was measured at 450 nm. An amount of 1000× DMSO stock solution was prepared for each concentration of extract and diluted 1000 times in the culture media to achieve the indicated concentrations. APAP was prepared as 200× stock in DMSO and spiked to the culture media to achieve the indicated concentrations. Where APAP or extract stock solution was not treated, the same amount of DMSO was spiked (final DMSO concentration 0.6%). All measurements were performed in triplicate.

### 3.5. Gas Chromatography-Mass Spectrometry

The GC-MS analysis of AAs and PAs was performed using an Agilent 7890 N gas chromatograph (Agilent Technologies, Santa Clara, CA, USA) interfaced with an Agilent 5975C mass-selective detector (70 eV, electron ionization mode) using an Ultra-2 capillary column (25 m × 0.20 mm I.D., 0.11 μm film thickness; Agilent Technologies, Santa Clara, CA, USA). Helium was used as the carrier gas at a constant flow rate of 0.5 mL/min. Samples (1 μL) were then injected and the analysis was conducted in split-injection mode (10:1). For amino acid analysis, the oven temperature was set initially to 140 °C for 2 min and increased to 240 °C at a 5 °C/min rate, and finally to 300 °C at a 30 °C/min rate with a holding time of 5 min. For phenolic acid analysis, the oven temperature was set initially at 180 °C for 1 min and increased to 300 °C at a 15 °C/min rate with a holding time of 5 min.

### 3.6. Sample Preparation for Amino Acids Profiling Analysis

AA profiling analysis (as EOC-TBDMS derivatives) was performed using GC-MS as described in a previous study [21]. Each Lycii Fructus sample (0.1 mg) with norvaline as internal standard (IS) (0.1 μg) were spiked into deionized water (1 mL) and added to dichloromethane (2.0 mL) containing ECF (20 μL), after which the pH was adjusted to ≥12 with 5.0 M sodium hydroxide. A two-phase EOC reaction was performed via vortex mixing for 10 min. After the EOC reaction, the pH was adjusted to ≤2 with 10% H_2_SO_4_, after which the product was saturated with sodium chloride and sequentially extracted using diethyl ether (3 mL) and ethyl acetate (2 mL). The extracts were evaporated to dryness under a gentle stream of nitrogen (40 °C). Prior to GC-MS analysis, the TBDMS derivative was produced with toluene (15 μL), MTBSTFA (20 μL), and triethylamine (5 μL) for 30 min at 60 °C. All samples were prepared individually in triplicate and analyzed directly by GC-MS.

### 3.7. Sample Preparation for Phenolic Acids Profiling Analysis

PA profiling analysis (as TBDMS derivatives) was performed using GC-MS [22,23]. Each Lycii Fructus sample (10 mg) with naproxen as an internal standard (0.2 μg) was spiked into deionized water (1 mL) and was adjusted to pH ≤ 2 with 10% H_2_SO_4_, saturated with sodium chloride, and sequentially extracted three times using diethyl ether and a dichloromethane mixture (2:1, *v*:*v*). The extracts were evaporated to dryness under a gentle stream of nitrogen (40 °C). Prior to GC-MS analysis, the TBDMS derivative was produced with toluene (15 μL), MTBSTFA (20 μL), and triethylamine (5 μL) for 60 min at 100 °C All samples were prepared individually in triplicate and analyzed directly by GC-MS.

### 3.8. Star Symbol Plotting

The levels of 24 AAs and nine PAs in each Lycii Fructus were determined based on calibration curves. The mean AA and PA values in *L. chinense* were normalized to the corresponding mean values of *L. barbarum*. Each normalized value was plotted as a line radiating from a common central point. The far ends of the lines were joined together to produce star symbol plots using MS Excel (Microsoft, Redmond, WA, USA) [24,25].

### 3.9. HPLC Analysis

HPLC analysis was carried out on an Agilent 1200 HPLC system with a G1315C diode array detector and an Agilent Technologies 6120 quadrupole instrument using a reverse phased column (Phenomenex Luna C-18 (2) 100 Å, 100 × 4.6 mm, 5 μm). The column was maintained at 25 °C and the flow rate was set at 1.0 mL/min. A 15 μL volume was injected into the column. Mobile phases were water (solvent A) and CH_3_CN (solvent B) solutions containing 0.1% (*v*/*v*) TFA. The initial conditions were 95:5 (A:B) and were held for 2 min; the percentage of CH_3_CN was increased to 30 from 2 to 10 min and held for 7 min; the percentage of CH_3_CN was increased linearly from 17 to 20 min and held for 3 min.

### 3.10. NMR Analysis

NMR spectra were recorded on 300 and 400 MHz for ^1^H-NMR, and 100 and 125 MHz for ^13^C-NMR (Varian Inova, Agilent, Santa Clara, CA, USA). CD_3_OD was used for the NMR solvent (Cambridge Isotope Laboratories, Tewksbury, MA, USA).

## 4. Conclusions

*L. chinense* and *L. barbarum* are currently discriminated based on gustatory patterns and sweetness [9]. However, this method is not suitable to identify species or origin in the health food or drink industries, which employ the herbs in powder or extract forms. Also, recently developed discrimination methods have limitations for the analysis of processed herb products. The star symbol plots produced from the analysis of 24 AAs and 9 PAs conducted herein were unique for both species and could therefore be used to discriminate between them. Additionally, compounds **1** and **2** would be useful identification markers for the discrimination of these two species. Importantly, the hepatoprotective activity of compound **1** partially demonstrated the therapeutic values of the *L. chinense*.

## Figures and Tables

**Table 1:** Amino acid levels in *L. chinense* and *L. barbarum*.

No.	Amino Acid	Retention Time	Selected Ions ^1^ (*m*/*z*)			Amount (ng/mg, Mean ± SD)		Normalized Value ^2^
						*L. barbarum*	*L. chinense*	
1	Alanine	5.17	190	**218**	260	2187.6 ± 67.8	565.2 ± 13.6	0.26
2	Glycine	5.40	176	**204**	246	36.5 ± 1.1	132.9 ± 1.0	3.64
3	α-Aminobutyric acid	6.06	130	204	**232**	12.9 ± 0.3	29.2 ± 0.6	2.27
4	Valine	6.51	144	218	**246**	290.3 ± 10.4	412.8 ± 15.2	1.42
5	Leucine	7.37	158	232	**260**	234.1 ± 8.0	683.4 ± 7.2	2.92
6	Isoleucine	7.62	158	232	**260**	55.7 ± 1.5	400.7 ± 5.7	7.19
7	Proline	8.09	142	216	**244**	9798.5 ± 242.4	2931.3 ± 106.0	0.30
8	γ-Aminobutyric acid	8.12	186	201	**346**	121.4 ± 4.2	1108.0 ± 31.3	9.13
9	Pipecolic acid	8.81	156	230	**258**	61.0 ± 1.7	97.6 ± 5.0	1.60
10	Pyroglutamic acid	10.25	272	**300**	342	653.7 ± 20.6	240.0 ± 8.7	0.37
11	Serine	11.03	216	302	**348**	514.0 ± 59.5	530.8 ± 42.9	1.03
12	Threonine	11.12	**230**	248	261	159.6 ± 33.2	146.2 ± 17.7	0.92
13	Phenylalanine	12.17	195	205	**294**	39.1 ± 1.7	440.7 ± 17.9	11.28
14	Cysteine	12.58	216	276	**322**	157.8 ± 0.5	158.7 ± 0.3	1.01
15	Aspartic acid	12.81	287	330	287	330	1079.9 ± 24.0	0.85
16	*N*-Methyl-DL-aspartic acid	13.01	244	287	**390**	40.5 ± 1.7	23.1 ± 0.5	0.57
17	Homocysteine	13.85	234	290	**336**	81.2 ± 7.6	56.5 ± 0.3	0.70
18	Glutamic acid	14.18	288	344	**390**	346.2 ± 15.3	559.5 ± 31.9	1.62
19	Asparagine	14.47	286	329	**375**	3460.4 ± 146.9	15211.9 ± 1141.6	4.40
20	α-Aminoadipic acid	15.36	302	358	**404**	33.6 ± 0.1	39.4 ± 0.7	1.17
21	Glutamine	15.71	287	343	**389**	7462.7 ± 588.4	1468.8 ± 119.1	0.20
22	Lysine	15.85	**156**	301	347	185.9 ± 6.1	897.2 ± 57.3	4.83
23	Histidine	16.35	254	267	**356**	565.5 ± 9.6	1441.7 ± 238.2	2.55
24	Tryptophan	18.35	130	**244**	333	156.8 ± 3.9	520.5 ± 118.1	3.32

^1^ The bolded ions were used for quantification. ^2^ Normalized value = amino acid amount of *L. chinense*/amino acid amount of *L. barbarum.*

**Table 2:** Phenolic acid levels in *L. chinense* and *L. barbarum*.

No.	Phenolic Acid	Retention Time	Selected Ions ^1^ (*m*/*z*)			Amount (ng/mg, Mean ± SD)		Normalized Value ^2^
						*L. barbarum*	*L. chinense*	
1	Salicylic acid	4.44	**73**	115	195	1.8 ± 0.1	2.3 ± 0.4	1.23
2	4-Hydroxybenzoic acid	5.27	73	235	**285**	8.1 ± 0.3	7.8 ± 0.1	0.96
3	Vanillic acid	6.02	193	223	**267**	1.8 ± 0.1	26.4 ± 0.3	14.81
4	Syringic acid	6.74	223	253	**297**	0.3 ± < 0.1	0.9 ± < 0.1	2.97
5	*p*-Coumaric acid	7.10	73	**261**	291	178.4 ± 9.3	6.8 ± < 0.1	0.04
6	Protocatechuic acid	7.47	**73**	365	395	1.0 ± < 0.1	0.7 ± < 0.1	0.72
7	Ferulic acid	7.86	219	249	**293**	33.6 ± 3.6	31.3 ± 0.3	0.93
8	Gallic acid	8.88	**73**	323	439	1.2 ± 0.1	1.9 ± 0.3	1.55
9	Caffeic acid	9.08	**73**	219	261	2.5 ± 0.3	0.7 ± 0.1	0.29

^1^ The bolded ions were used for quantification. ^2^ Normalized value = phenolic acid amount of *L. chinense*/phenolic acid amount of *L. barbarum.*

## Supplementary Materials

The following materials are available online. ^1^H- and ^13^C-NMR spectra of compounds **1** and **2** (Figures S1–S4), commercially available Lycii Fructus, and corresponding LC chromatograms (Figures S5 and S6).
