# Supplementary material for: Discrimination of Lycium chinense and L. barbarum Based on Metabolite Analysis and Hepatoprotective Activity
Source: Molecules. 2020 Dec 10;25(24):5835. doi: 10.3390/molecules25245835 (PMC7764731; doi:10.3390/molecules25245835)

Supporting Information for

Discrimination of *Lycium chinense* and *L. barbarum* Based on Metabolite Analysis and Hepatoprotective Activity

Min-Ji Ryu[1,#], Minjeong Kim[2,#], Moongi Ji[3,#], Chaeyoung Lee[2], Inho Yang[4], Seong-Bin Hong[5], Jungwook Chin[6], Eun Kyoung Seo[2], Man-Jeong Paik[3,*], Kyung-Min Lim[2,*] and Sang-Jip Nam[1,*]

[1] Department of Chemistry and Nanoscience, Ewha Womans University, Seoul 03760, Korea; ryumj624@naver.com (M.-J.R.) sjnam@ewha.ac.kr (S.-J.N.)

[2] College of Pharmacy, Ewha Womans University, Seoul 03760, Korea; nabe37@naver.com (M.K.) chaeyoung510@gmail.com (C.L.) yuny@ewha.ac.kr (E.K.S.) kmlim@ewha.ac.kr (K.-M.L.)

[3] College of Pharmacy, Sunchon National University, Suncheon 57922, Korea; wlansrl@naver.com (M.J.) paik815@scnu.ac.kr (M.-J.P.)

[4] Department of Convergence Study on the Ocean Science and Technology, Korea Maritime and Ocean University, Busan 4912, Korea; ihyang@kmou.ac.kr

[5] Biomix Co., Ltd. 142, Ilsan-ro, Ilsandong-gu, Goyang-si, Gyeonggi-do 10442, Korea; au4030@hanmail.net

[6] New Drug Development Center, Daegu Gyeongbuk Medical Innovation Foundation, Daegu 41061, Korea; jwchin@dgmif.re.kr

[#] These authors contributed equally to this work.

* Correspondence: paik815@scnu.ac.kr (M.-J.P.), kmlim@ewha.ac.kr (K.-M.L.), sjnam@ewha.ac.kr (S.-J.N.)

Table of Contents

Figure S1. ^{1}H NMR spectrum of 4-[formyl-5-(hydroxymethyl)-1*H*-pyrrol-1-yl]butanoic acid (**1**) in CD_3OD-d_4

Figure S2. ^{13}C NMR spectrum of 4-[formyl-5-(hydroxymethyl)-1*H*-pyrrol-1-yl]butanoic acid (**1**) in CD_3OD-d_4

Figure S3. ^{1}H NMR spectrum of *p*-coumaric acid (**2**) in CD_3OD-d_4

Figure S4. ^{13}C NMR spectrum of *p*-coumaric acid (**2**) in CD_3OD-d_4

Figure S5. Commercially available Lycii Fructus of *L.chinense* and *L. barbarum*. (12 samples of two *Lycium* spp.)

Figure S6. LC Chromatogram of Lycii Fructus of *L.chinense* and *L. barbarum*. Numbers corresponding sample number of Figure S5. Each peaks were identified as 4-(2-formyl-5-(hydroxymethyl)-1*H*-pyrrol-1-yl)butanoic acid (**1**) and *p*-coumaric acid (**2**).

Figure S1. ^{1}H NMR spectrum of 4-[formyl-5-(hydroxymethyl)-1*H*-pyrrol-1-yl]butanoic acid (**1**) in CD_3OD-*d*$_4$.

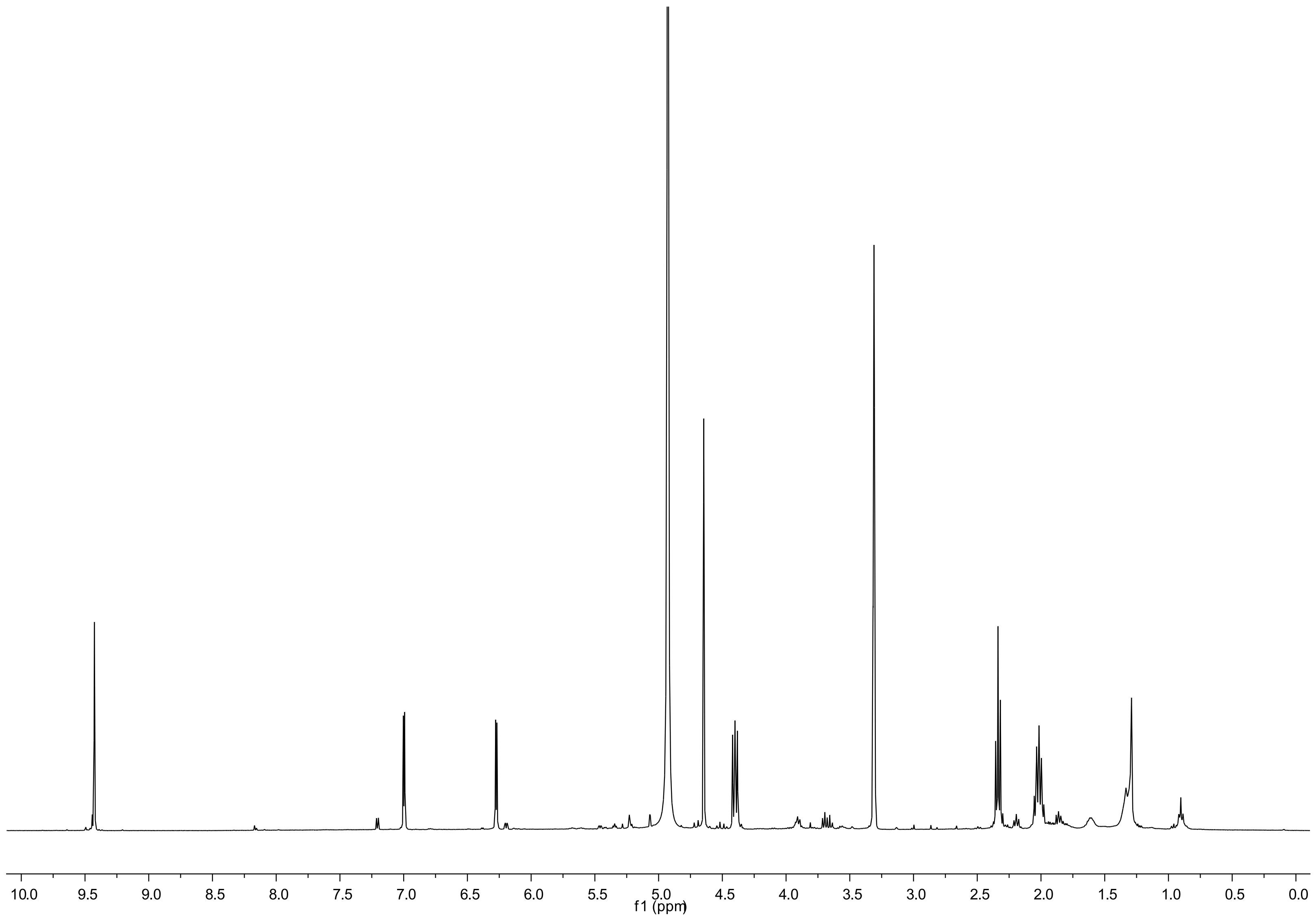

Figure S2. ^{13}C NMR spectrum of 4-[formyl-5-(hydroxymethyl)-1*H*-pyrrol-1-yl]butanoic acid (**1**) in CD_3OD-*d*$_4$

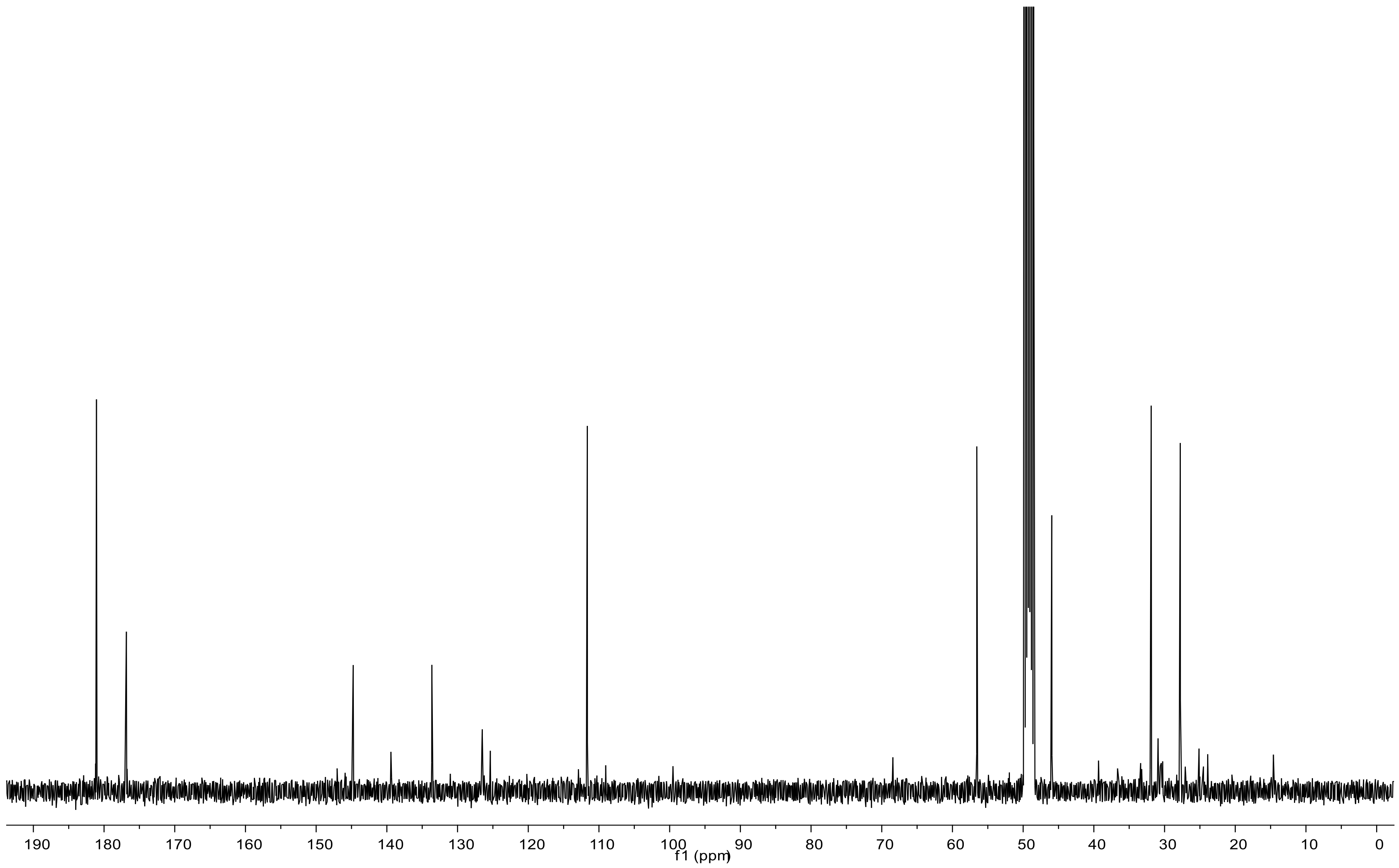

Figure S3. ^{1}H NMR spectrum of *p*-coumaric acid (**2**) in CD_3OD-d_4

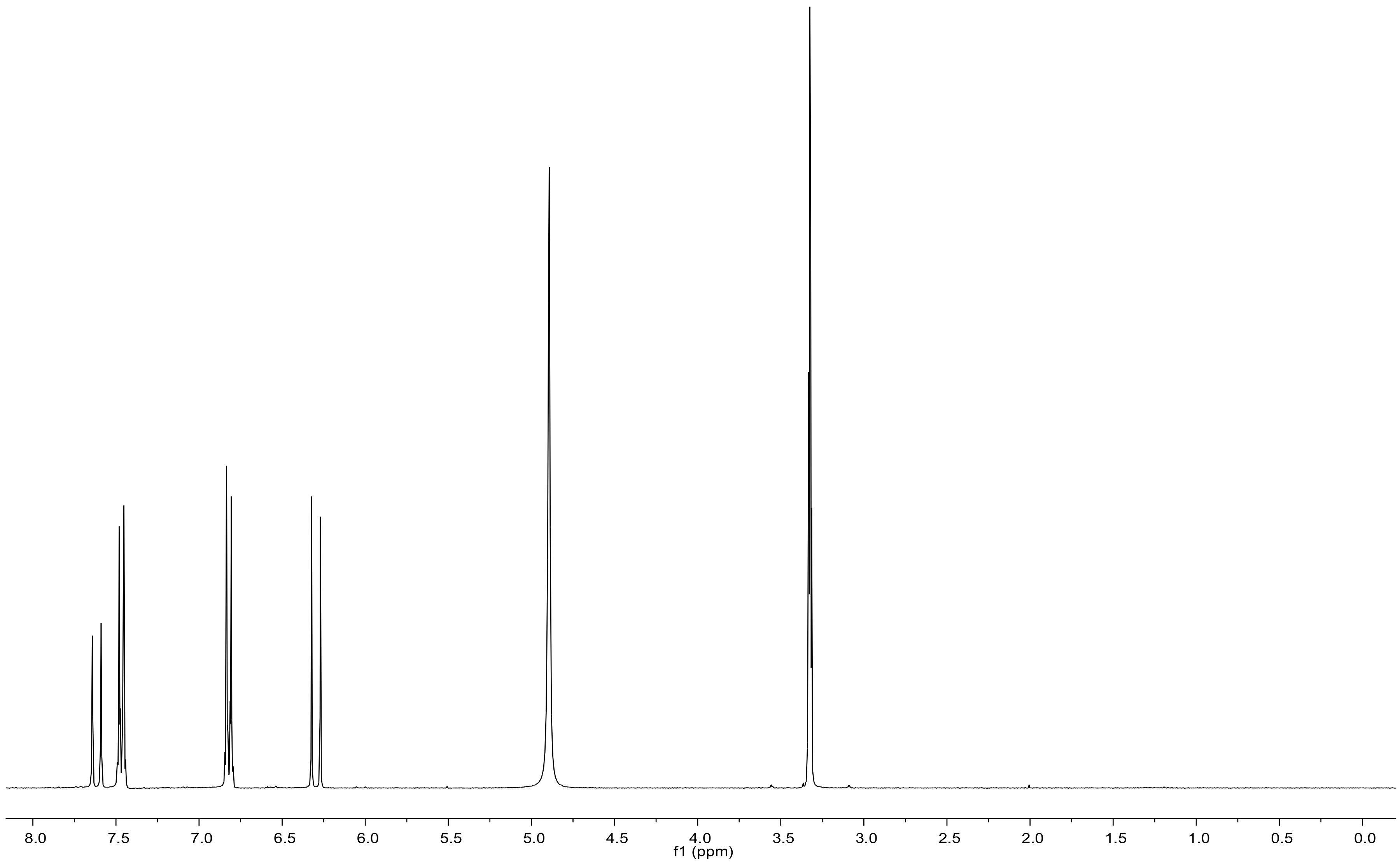

Figure S4. ^{13}C NMR spectrum of *p*-coumaric acid (**2**) in CD_3OD-d_4

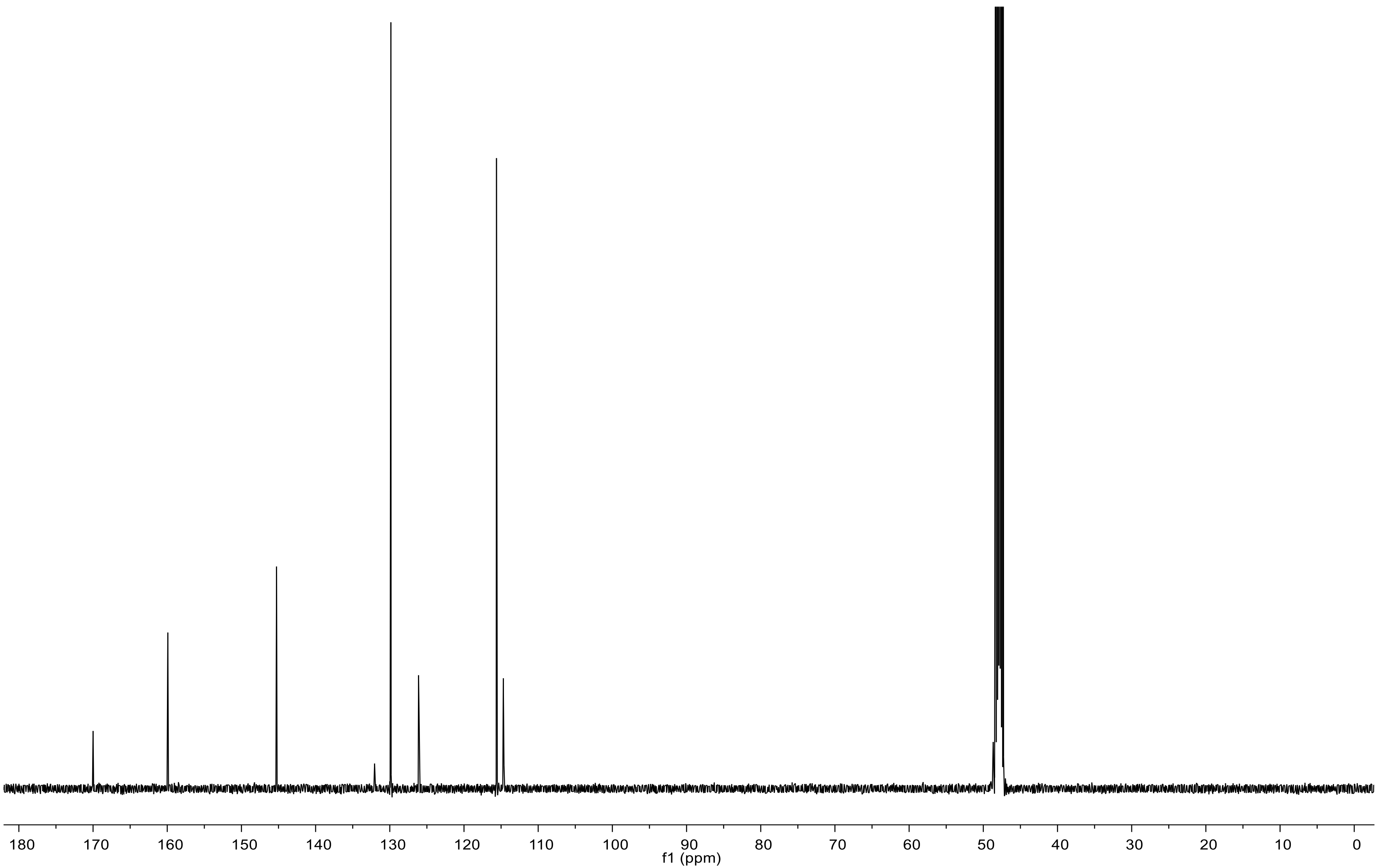

Figure S5. Commercially available Lycii Fructus of *L.chinense* and *L. barbarum*. (12 samples of two *Lycium* spp.)

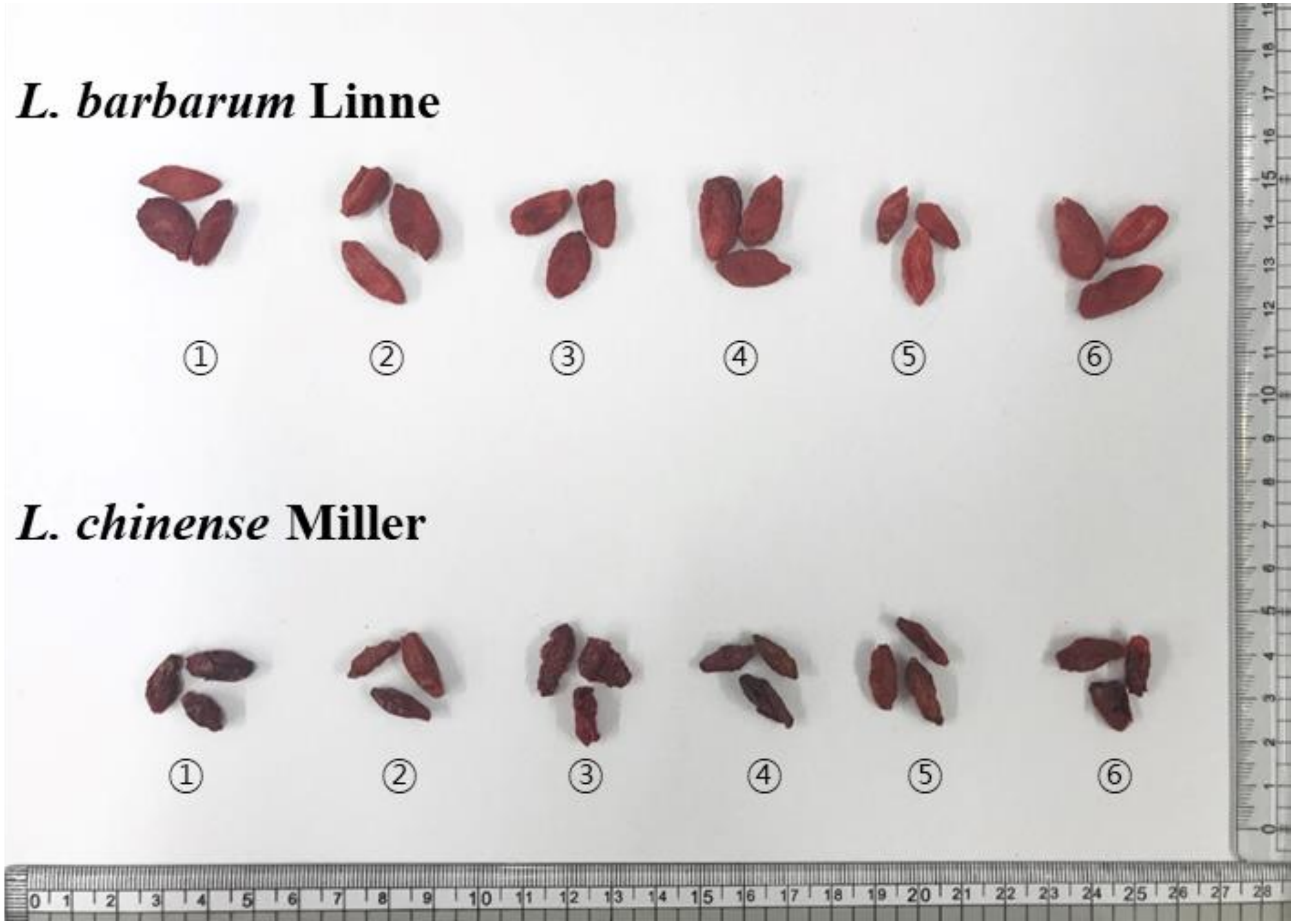

Figure S6. LC Chromatogram of Lycii Fructus of *L.chinense* and *L. barbarum*. Numbers corresponding sample number of Figure S5. Each peaks were identified as 4-(2-formyl-5-(hydroxymethyl)-1*H*-pyrrol-1-yl)butanoic acid (**1**) and *p*-coumaric acid (**2**).

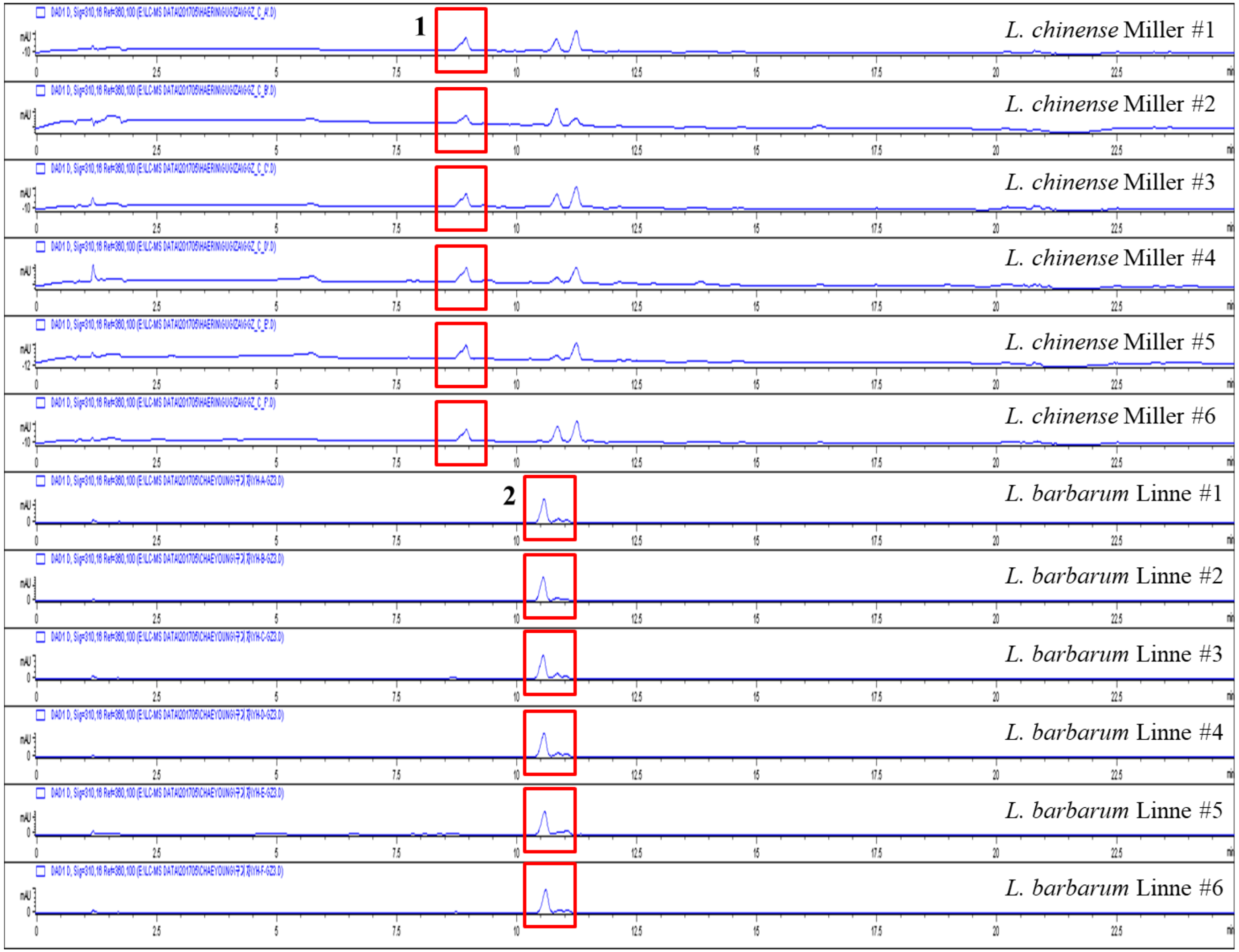

Supplement: Supplementary file 1 [file molecules-25-05835-s001.pdf]
